# Single Agent Antihypertensive Therapy and Orthostatic Blood Pressure Behaviour in Older Adults Using Beat-to-Beat Measurements: The Irish Longitudinal Study on Ageing

**DOI:** 10.1371/journal.pone.0146156

**Published:** 2016-01-05

**Authors:** Mark Canney, Matthew D. L. O’Connell, Catriona M. Murphy, Neil O’Leary, Mark A. Little, Conall M. O’Seaghdha, Rose Anne Kenny

**Affiliations:** 1 The Irish Longitudinal Study on Ageing, Trinity College Dublin, Dublin, Ireland; 2 Trinity Health Kidney Centre, Trinity College Dublin, Dublin, Ireland; 3 Department of Renal Medicine, Beaumont Hospital, Dublin, Ireland; Universita degli Studi di Napoli Federico II, ITALY

## Abstract

**Background:**

Impaired blood pressure (BP) stabilisation after standing, defined using beat-to-beat measurements, has been shown to predict important health outcomes. We aimed to define the relationship between individual classes of antihypertensive agent and BP stabilisation among hypertensive older adults.

**Methods:**

Cross-sectional analysis from The Irish Longitudinal Study on Ageing, a cohort study of Irish adults aged 50 years and over. Beat-to-beat BP was recorded in participants undergoing an active stand test. We defined grade 1 hypertension according to European Society of Cardiology criteria (systolic BP [SBP] 140-159mmHg ± diastolic BP [DBP] 90-99mmHg). Outcomes were: (i) initial orthostatic hypotension (IOH) (SBP drop ≥40mmHg ± DBP drop ≥20mmHg within 15 seconds [s] of standing accompanied by symptoms); (ii) sustained OH (SBP drop ≥20mmHg ± DBP drop ≥10mmHg from 60 to 110s inclusive); (iii) impaired BP stabilisation (SBP drop ≥20mmHg ± DBP drop ≥10mmHg at any 10s interval during the test). Outcomes were assessed using multivariable-adjusted logistic regression.

**Results:**

A total of 536 hypertensive participants were receiving monotherapy with a renin-angiotensin-aldosterone-system inhibitor (n = 317, 59.1%), beta-blocker (n = 89, 16.6%), calcium channel blocker (n = 89, 16.6%) or diuretic (n = 41, 7.6%). A further 783 untreated participants met criteria for grade 1 hypertension. Beta-blockers were associated with increased odds of initial OH (OR 2.05, 95% CI 1.31–3.21) and sustained OH (OR 3.36, 95% CI 1.87–6.03) versus untreated grade 1 hypertension. Multivariable adjustment did not attenuate the results. Impaired BP stabilisation was evident at 20s (OR 2.59, 95% CI 1.58–4.25) and persisted at 110s (OR 2.90, 95% CI 1.64–5.11). No association was found between the other agents and any study outcome.

**Conclusion:**

Beta-blocker monotherapy was associated with a >2-fold increased odds of initial OH and a >3-fold increased odds of sustained OH and impaired BP stabilisation, compared to untreated grade 1 hypertension. These findings support existing literature questioning the role of beta-blockers as first line agents for essential hypertension.

## Introduction

The optimum treatment strategy for hypertension in older individuals is controversial. Although blood pressure (BP) reduction has been shown to improve cardiovascular outcomes even in the very old [1], the use of multiple antihypertensive agents to achieve a systolic BP below 130mmHg has been demonstrated to increase the risk of mortality in vulnerable older adults [2]. The observation that aggressive BP lowering may be hazardous among older adults has been acknowledged by the recent introduction of a more conservative BP target of <150/90 mmHg for adults ≥60 years in the eighth report of the US Joint National Committee guidelines for the treatment of hypertension [3].

Orthostatic hypotension (OH) is a complex condition that is associated with substantial morbidity and mortality [4]. The prevalence of both hypertension and OH increase with age, resulting in a therapeutic dilemma termed “supine hypertension-orthostatic hypotension” [5]. The current consensus definition of OH is a sustained reduction in systolic blood pressure (SBP) of at least 20 mmHg or in diastolic blood pressure (DBP) of at least 10 mmHg within three minutes of standing [6]. In standard clinical practice the diagnosis of OH is made using an auscultatory or oscillometric BP measurement before standing and a second measurement within a three-minute window after standing, thus providing a limited number of data points from which to ascertain BP behaviour. Recently, continuous non-invasive, or “beat-to-beat”, BP measurements have been developed for clinical use. This technology has greatly enhanced our ability to interrogate postural changes in BP and has facilitated new approaches to the diagnosis of OH, in particular *sustained* orthostatic change [7]. Beat-to-beat data provide an illustration of BP behaviour during orthostasis, including the magnitude of the initial drop and the slope of BP recovery, or stabilisation, towards its baseline value. There is evidence that the degree of BP stabilisation, rather than the nadir value, is a predictor of important health outcomes in older individuals [8].

The differential effects of individual antihypertensive agents on the compensatory BP response after standing have not been formally assessed using beat-to-beat measurements. The aim of this study was to define the relationship between individual classes of antihypertensive agent and BP stabilisation during an active stand test. We hypothesised that antihypertensive agents would demonstrate differential BP responses to an orthostatic challenge. We tested this by measuring beat-to-beat BP during an active stand test in a population of older hypertensive adults who were receiving single agent antihypertensive therapy.

## Methods

### Study Population

Data from the first wave of The Irish Longitudinal Study on Ageing (TILDA) were analysed. TILDA is a nationally representative prospective cohort study of community-dwelling Irish adults aged 50 years and over. Details of the sampling procedure and study design have been described elsewhere [9, 10]. Wave 1 was performed between June 2009 and July 2011. All participants undertook a computer-assisted personal interview in their homes along with a self-completion questionnaire. Participants were subsequently invited to take part in a detailed health assessment in one of two dedicated health centres, carried out by trained nurses. Participants were not required to fast in advance of the health assessments, which were performed at staggered intervals throughout the day from 8.30am to 6pm. All participants provided informed signed consent and ethical approval for the study was granted by the Research Ethics Committee of Trinity College Dublin. All experimental procedures adhered to the Declaration of Helsinki.

**Fig 1:**
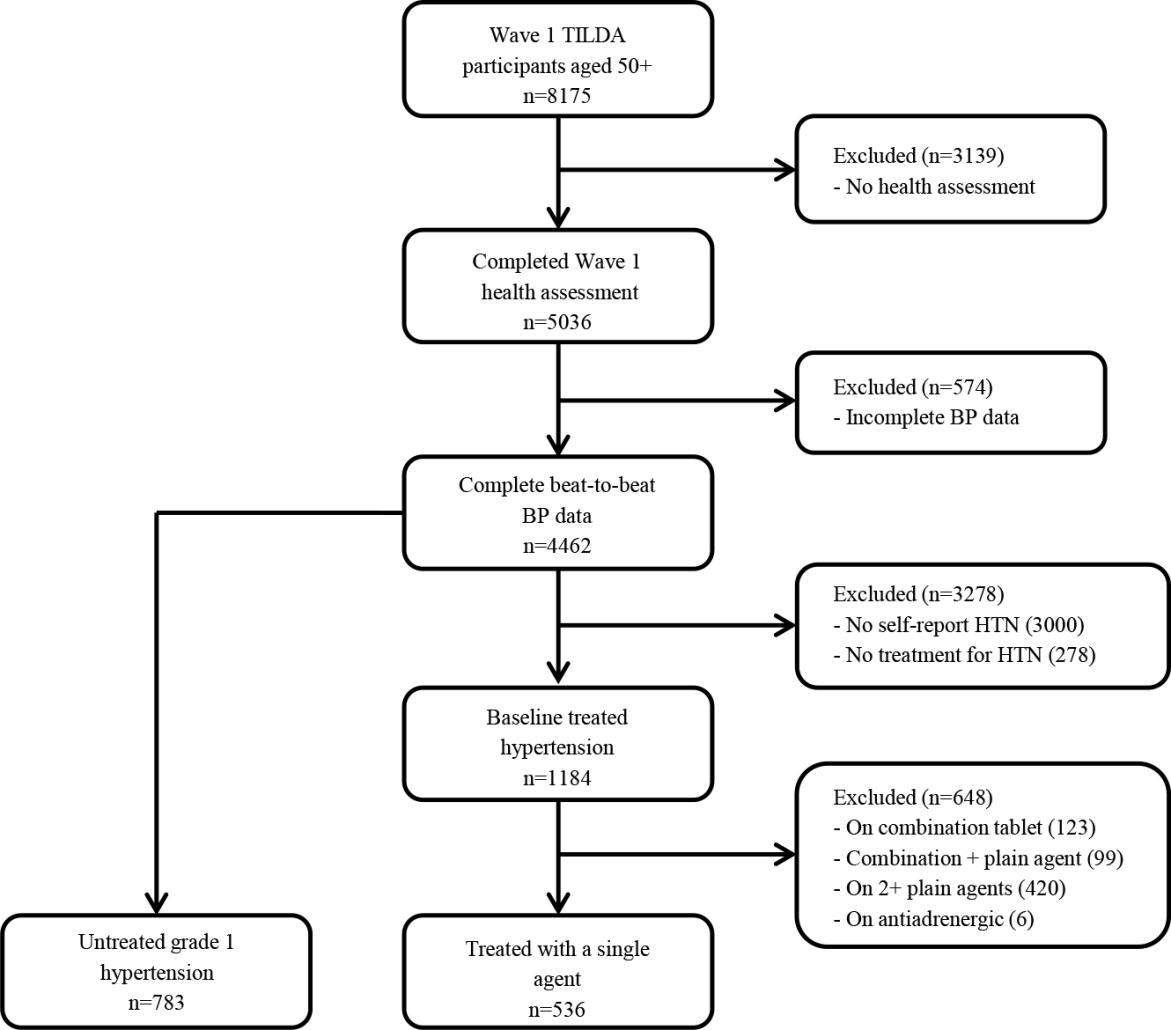
Flowchart of study population. Complete beat-to-beat BP data was available in 4462 participants of whom 1184 reported a physician’s diagnosis of hypertension (HTN) and were receiving treatment. A total of 536 participants were receiving single agent therapy with a renin-angiotensin-aldosterone-system inhibitor, beta-blocker, calcium channel blocker or diuretic. The reference group consisted of 783 participants with grade 1 hypertension who were not receiving any anti-hypertensive therapy.

Of the 8175 TILDA participants aged ≥ 50 years initially recruited, 5036 individuals (62%) took part in the wave 1 health assessment. Participants who did not complete a health assessment (n = 3139) tended to be older (mean age 66.8 vs 62 years) and to have a greater burden of co-morbid conditions. Complete beat-to-beat BP data during an active stand test were available for 4462 participants, of whom 1184 were receiving treatment for self-reported physician-diagnosed hypertension **(Fig 1)**. A total of 536 participants (45.3% of all treated hypertensive subjects) were receiving single agent antihypertensive therapy with a renin-angiotensin-aldosterone-system (RAAS) blocker (n = 317, 59.1%), beta-blocker (n = 89, 16.6%), calcium channel blocker (n = 89, 16.6%) or diuretic (n = 41, 7.6%). Participants taking multiple anti-hypertensive agents were excluded. Of the 574 participants with incomplete BP data, 84 (14.7%) were receiving single agent therapy for self-reported physician-diagnosed hypertension. Compared to the final study population (n = 536), these 84 individuals were, on average, 3 years older (66.4 vs 63.1 years).

### Study Outcomes

We chose three outcomes for this study: (i) initial OH, (ii) sustained OH and (iii) impaired BP stabilisation. We defined initial OH as a drop in SBP of ≥ 40mmHg or a drop in DBP of ≥ 20mmHg within the first 15 seconds of standing accompanied by symptoms. We defined sustained OH as a drop that exceeded consensus BP thresholds for OH (SBP drop ≥ 20mmHg or DBP drop ≥ 10mmHg) at all of the following time points (T): 60, 70, 80, 90, 100, and 110 seconds after standing [7]. Impaired BP stabilisation was recorded at each 10 second interval and was defined as OH (SBP drop ≥ 20mmHg or DBP drop ≥ 10mmHg) at any time point (T = 10, 20, 30,…, 110 seconds).

The BP response to postural change was recorded using the volume-clamp method combined with Physiocal and brachial artery waveform reconstruction (Finometer, Finapres Medical Systems, Arnhem, Netherlands). Recordings were obtained in a comfortably lit quiet room at ambient temperature (21 to 23°C). Participants were asked to rest in the supine position for ten minutes. Throughout this time the Physiocal (recalibration) function was enabled. Baseline SBP and DBP values were calculated as the mean SBP and DBP measurements recorded between 60 and 30 seconds prior to the participant standing. These BP measurements were used to define the reference group. Physiocal was switched off immediately prior to standing and remained off until completion of the active stand test, to ensure that no data were lost during this time period due to the recalibration process. Participants were asked to stand in a timely manner (less than 5 seconds) with or without assistance from the research nurse. Upon standing SBP and DBP were recorded for 2 minutes, during which time the participants stood quietly. The participants’ BP was estimated at 10-second intervals using 5-second moving averages around each time point. Subjects were asked to report symptoms such as dizziness during the test (orthostatic intolerance).

### Predictor variables

Antihypertensive medications were coded according to the World Health Organisation Anatomical Therapeutic Chemical Classification (ATC) [11]. For the purposes of this analysis, four classes of anti-hypertensive medication were considered: (i) RAAS blockers, including angiotensin converting enzyme inhibitors (ATC C09A), angiotensin II receptor blockers (ATC C09C) and direct renin inhibitors (ATC C09X); (ii) beta-blockers (ATC C07A); (iii) calcium channel blockers (ATC C08C, C08D, C08E); (iv) diuretics (ATC C03). Medication use was recorded during the interview and cross-checked with medication labels. Richardson *et al* previously demonstrated a good level of agreement (kappa scores 0.77–0.80) between interview-ascertained medication use with these four classes of antihypertensive agent and pharmacy records in the TILDA sample [12].

### Covariates

Characteristics recorded at wave 1 included age, gender, educational attainment (primary, secondary, tertiary), smoking history, and self-reported physician-diagnosed conditions. Body mass index (BMI) was calculated at the health assessment. Serum low density lipoprotein (LDL), high density lipoprotein (HDL), and total cholesterol were measured from participants’ blood taken at the wave 1 health assessment. We defined cardiovascular disease as any one of the following conditions: angina, myocardial infarction, coronary artery stenting, coronary artery bypass surgery, heart failure, arrhythmia or stroke. We identified the following variables as cardiovascular risk factors: diabetes mellitus, current smoking, BMI, LDL and HDL cholesterol. The use of anti-depressant medication (ATC N06A) was chosen as a covariate based on previous literature demonstrating its association with OH [5]. Anti-psychotics (ATC N05A) and benzodiazepines (ATC N05BA, N05CD, N05CF) were also considered potential confounding variables and were incorporated into the regression models as a pooled covariate called “other psychotropic medication.”

### Statistical Analysis

Normally distributed continuous variables were described as means and standard deviations (SD) and compared across drug categories using ANOVA. Non-normally distributed continuous variables were described as medians and interquartile ranges (IQR) and compared using Kruskal-Wallis test. Categorical variables were compared using the chi-square test or Fisher’s exact test as appropriate. Study outcomes were assessed using logistic regression models. The reference group for the regression models consisted of participants with objective evidence of grade 1 hypertension who were not receiving any anti-hypertensive therapy (n = 783). We defined grade 1 hypertension according to the European Society of Cardiology criteria (SBP 140-159mmHg and/or DBP 90-99mmHg) [13]. Three logistic regression models were constructed: (1) adjusted for age and sex; (2) adjusted for model 1 covariates plus baseline SBP; (3) adjusted for model 2 covariates plus educational attainment, use of anti-depressants, use of other psychotropic medication, cardiovascular disease and cardiovascular risk factors.

To further illustrate the relationship between antihypertensive medications and orthostatic BP behaviour, the mean differences in systolic and diastolic BP values from baseline at each 10-second interval were estimated from linear regression models and plotted for each of the antihypertensive drug groups and the reference group. These linear models were adjusted for all covariates included in model 3 above. Additionally, we performed a sensitivity analysis of the association between antihypertensive therapy and sustained OH by excluding participants with self-reported cardiovascular disease from the study population. The logistic regression models performed in this analysis were the same as those described above, except for model 3 which did not include the cardiovascular disease variable.

In a secondary analysis we performed pairwise comparisons to directly compare the odds ratios of IOH, sustained OH and OH at 30, 60, 90 and 110 seconds among the four antihypertensive drug classes. These comparisons were conducted on the fully adjusted logistic regression model (model 3). In order to reduce the risk of type 1 error from multiple testing, we employed the Sidak correction method for all pairwise comparisons. All analyses were performed using Stata version 12.1 (StataCorp, College Station, TX). All tests were two-tailed at an alpha level of 0.05.

## Results

### Characteristics of the Study Population

Baseline characteristics of the study population are shown in **Table 1**. Among the drug categories the calcium channel blocker group tended to be older (mean age [± SD] 64.8 ± 8.1 years) and the diuretic group tended to be younger (62.5 ± 8.1 years). The diuretic group had a high proportion of female participants (75%). Baseline mean SBP (± SD) tended to be higher in the calcium channel blocker group (149.6 ± 23.9 mmHg) and lower in the RAAS blocker group (136.7 ± 23.2 mmHg). The prevalence of diabetes mellitus was highest in the RAAS blocker group (13.3%) and this group also had the highest median BMI (30.2 kg/m^2^, IQR 27.3–33.4). Pre-existing cardiovascular disease was more common in the beta-blocker group. Results for LDL and HDL cholesterol were unavailable in 37 out of 1319 participants (2.8%).

**Table 1:** Characteristics of the study population.

Characteristic	Untreated† (n = 783)	RAAS blocker (n = 317)	Beta-blocker (n = 89)	CCB (n = 89)	Diuretic (n = 41)	p-value††
Age in years, mean (SD)	60.8 (7.6)	62.8 (7.7)	62.8 (7.7)	64.6 (8.1)	62.5 (8.1)	0.3
Categorical age, n (%)						0.6
• 50 to 64 years	551 (70.4)	185 (58.4)	55 (61.8)	44 (49.4)	25(61.0)	
• 65 to 74 years	193 (24.7)	111 (35.0)	26 (29.2)	36 (40.5)	14(34.1)	
• 75 years and over	39 (5.0)	21 (6.6)	8 (9.0)	9 (10.1)	*2 (4*.*9)*	
Female sex, n (%)	431 (55.0)	162 (51.1)	41 (46.1)	54 (60.7)	31 (75.6)	0.006
3^rd^ level education or higher, n (%)	304 (38.8)	102 (32.2)	33 (37.1)	29 (32.6)	17 (41.5)	0.9
Baseline SBP (mmHg), mean (SD)	148.5 (5.8)	136.7 (23.2)	142.9 (22.8)	149.6 (23.9)	142.4 (20.9)	<0.0001
Baseline DBP (mmHg), mean (SD)	79.0 (7.5)	73.7 (11.7)	74.1 (9.8)	77.4 (13.2)	75.2 (10.0)	0.06
Diabetes mellitus, n (%)	32 (4.1)	42 (13.3)	4 (4.5)	2 (2.3)	3 (7.3)	0.004
Diabetes complications‡, n (%)	5 (15.6)	14 (33.3)	1 (25.0)	1 (50.0)	2 (66.7)	0.6
Current smoker, n (%)	111 (14.2)	39 (12.3)	15 (16.9)	9 (10.1)	3 (7.3)	0.4
Psychotropic medications, n (%)						
• Antidepressants	39 (5.0)	17 (5.4)	8 (9.0)	10 (11.2)	6 (14.6)	0.07
• Antipsychotics	3 (0.4)	2 (0.6)	1 (1.1)	0	0	0.8
• Benzodiazepines	26 (3.3)	16 (5.1)	6 (6.7)	9 (10.1)	2 (4.9)	0.4
BMI (kg/m^2^), median (IQR)	27.7 (25.2–30.6)	30.2 (27.3–33.4)	28.0 (26.6–30.7)	28.6 (25.5–32.2)	28.9 (26.2–33.1)	0.001
Total cholesterol (mmol/L), mean (SD)	5.42 (0.99)	4.80 (0.97)	4.73 (0.99)	5.09 (0.96)	4.93 (0.90)	0.05
HDL cholesterol (mmol/L), mean (SD)	1.60 (0.43)	1.46 (0.39)	1.40 (0.37)	1.60 (0.43)	1.57 (0.44)	0.002
LDL cholesterol (mmol/L), mean (SD)	3.16 (0.91)	2.61 (0.90)	2.61 (0.99)	2.84 (0.85)	2.86 (0.90)	0.09
Pre-existing CVD, n (%)						
• None	736 (94.0)	266 (83.9)	54 (60.7)	81 (91.0)	38 (92.7)	<0.001
• Angina	9 (1.2)	15 (4.7)	12 (13.5)	1 (1.1)	1 (2.4)	0.003
• Myocardial infarction	7 (0.9)	12 (3.8)	8 (9.0)	1 (1.1)	1 (2.4)	0.07
• Stent/bypass	2 (0.3)	3 (1.0)	4 (4.5)	0	0	0.06
• Heart failure	0	4 (1.3)	1 (1.1)	0	0	0.9
• Arrhythmia	36 (4.6)	22 (6.9)	19 (21.4)	5 (5.6)	2 (4.9)	0.001
• Stroke	2 (0.3)	9 (2.8)	3 (3.4)	2 (2.3)	1 (2.4)	1.0

BMI, body mass index; CCB, calcium channel blocker; CVD, cardiovascular disease; DBP, diastolic blood pressure; HDL, high density lipoprotein; LDL, low density lipoprotein; RAAS, renin-angiotensin-aldosterone-system; SBP, systolic blood pressure.

† Reference group consisted of untreated participants with grade 1 hypertension

†† For differences across the four drug categories

‡ Diabetes complications included self-reported physician-diagnosed leg ulcers, proteinuria, diabetic nephropathy, retinopathy and peripheral neuropathy. Numbers are expressed as a proportion of the total individuals with diabetes in each category.

### Study Outcomes

#### Initial orthostatic hypotension (IOH)

The prevalence of IOH was 27.7% in the total study population (n = 1319), 26.7% in the reference group (n = 783) and 29.1% in the treated population (n = 536). Participants on single agent therapy with a beta-blocker had the highest prevalence of IOH (42.7%). The unadjusted odds ratio for IOH in the beta-blocker group (compared to untreated grade 1 hypertension) was 2.05 (95% CI 1.31–3.21, p = 0.002), which was minimally attenuated after multivariable adjustment (Table 2). No significant association was found between IOH and the other three anti-hypertensive drug categories.

**Table 2:** Odds ratios (95% confidence intervals) of initial orthostatic hypotension and sustained orthostatic hypotension for each class of antihypertensive agent.

Outcome	Untreated†	RAAS blocker	Beta-blocker	CCB	Diuretic
**Initial OH**					
Unadjusted	*ref*	1.06 (0.79–1.41)	2.05 (1.31–3.21)**	0.90 (0.54–1.50)	0.67 (0.30–1.46)
Model 1	*ref*	1.06 (0.79–1.42)	2.02 (1.29–3.18)**	0.94 (0.56–1.57)	0.72 (0.32–1.58)
Model 2	*ref*	1.07 (0.78–1.46)	2.03 (1.29–3.20)**	0.94 (0.56–1.57)	0.72 (0.33–1.59)
Model 3	*ref*	1.14 (0.81–1.59)	1.97 (1.22–3.18)**	0.91 (0.54–1.55)	0.79 (0.35–1.77)
**Sustained OH**					
Unadjusted	*ref*	0.84 (0.49–1.45)	3.36 (1.87–6.03)**	1.49 (0.71–3.13)	n/a††
Model 1	*ref*	0.77 (0.45–1.33)	3.17 (1.75–5.75)**	1.21 (0.57–2.58)	n/a
Model 2	*ref*	0.88 (0.50–1.53)	3.33 (1.79–6.18)**	0.95 (0.43–2.12)	n/a
Model 3	*ref*	0.86 (0.45–1.62)	3.52 (1.79–6.93)***	1.10 (0.48–2.52)	n/a

CCB, calcium channel blocker; OH, orthostatic hypotension; RAAS, renin-angiotensin-aldosterone-system. Model 1 adjusted for age and sex. Model 2 adjusted for model 1 co-variates *plus* baseline systolic blood pressure. Model 3 adjusted for model 2 co-variates *plus* educational attainment, smoking, antidepressant use, other psychotropic medication, diabetes, body mass index, LDL and HDL cholesterol, and cardiovascular disease (any one of angina, myocardial infarction, coronary artery stenting, coronary artery bypass surgery, heart failure, arrhythmia or stroke).

† Reference group consisted of untreated participants with grade 1 hypertension

†† No events of sustained orthostatic hypotension were observed in the diuretic group

** p<0.01

***p<0.001

#### Sustained orthostatic hypotension

The overall prevalence of sustained OH was 7.7%. Among the 536 participants receiving single agent antihypertensive therapy, the prevalence of sustained OH was 8.6%. Participants on beta-blocker therapy had the highest prevalence of sustained OH (22%). No events of sustained OH were observed in the diuretic group. Odds ratios of sustained OH for each drug category are shown in Table 2. The unadjusted odds ratio for the beta-blocker group (versus untreated grade 1 hypertension) was 3.36 (95% CI 1.87–6.03), which was minimally attenuated after adjustment for age and sex (OR 3.17, 95% CI 1.75–5.75). Adjustment for baseline SBP and cardiovascular disease did not further attenuate the association. No association was found between sustained OH and the other drug categories. The proportion of participants with sustained OH who reported symptoms during the test differed by antihypertensive drug class. Half of individuals (50%) on beta-blocker therapy reported symptoms during orthostasis, compared to 32% on RAAS blockers, and 22% on calcium channel blockers.

#### Impaired BP stabilisation

**Fig 2:**
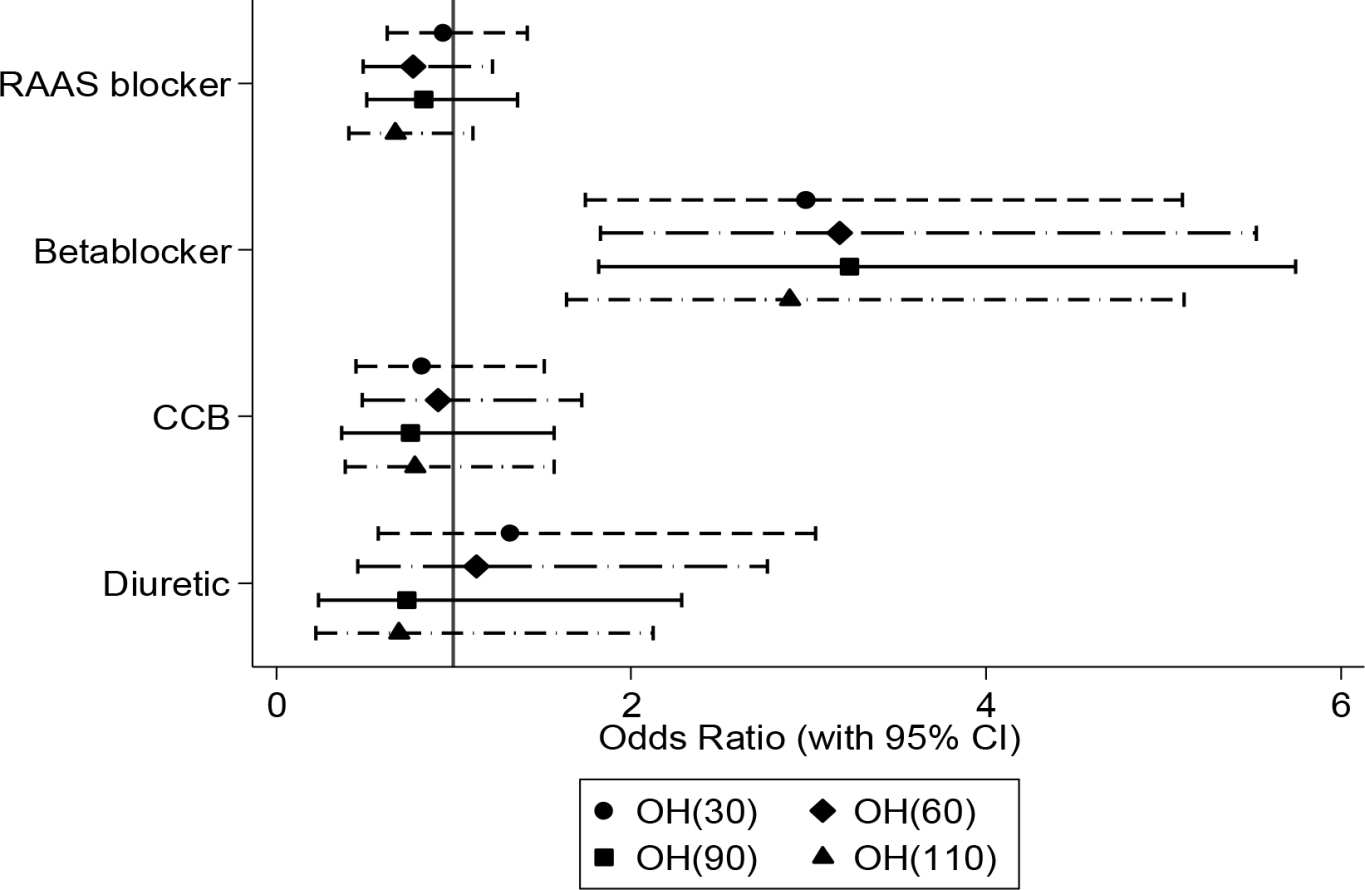
Plot of odds ratios (with 95% confidence interval) of orthostatic hypotension (OH) at 30, 60, 90 and 110 seconds post active stand for each antihypertensive agent. Each point estimate represents the fully adjusted odds of OH for a particular antihypertensive agent compared to the odds of OH for the reference group. Individuals receiving beta-blocker therapy had an approximately three-fold increased odds of OH at each time point compared to untreated participants. The odds ratios for the other three agents were not statistically significant (error bounds cross the reference line). CCB, calcium channel blocker; RAAS, renin-angiotensin-aldosterone-system.

The fully adjusted (model 3) odds ratios of OH at each 10-second interval for each drug category are shown in S1 Table and plotted in Fig 2 for the following time points: 30, 60, 90 and 110 seconds post active stand. The effect of beta-blockers was evident from 20 seconds after standing (OR 2.59, 95% CI 1.58–4.25) and persisted at each time point out to 110 seconds (OR 2.90, 95% CI 1.64–5.11). The peak effect of beta-blockers was observed between 50 seconds and 100 seconds, during which time a greater than three-fold increased odds of OH was demonstrated compared to untreated hypertensive participants. No consistent association was detected for the other three drug classes.

**Fig 3:**
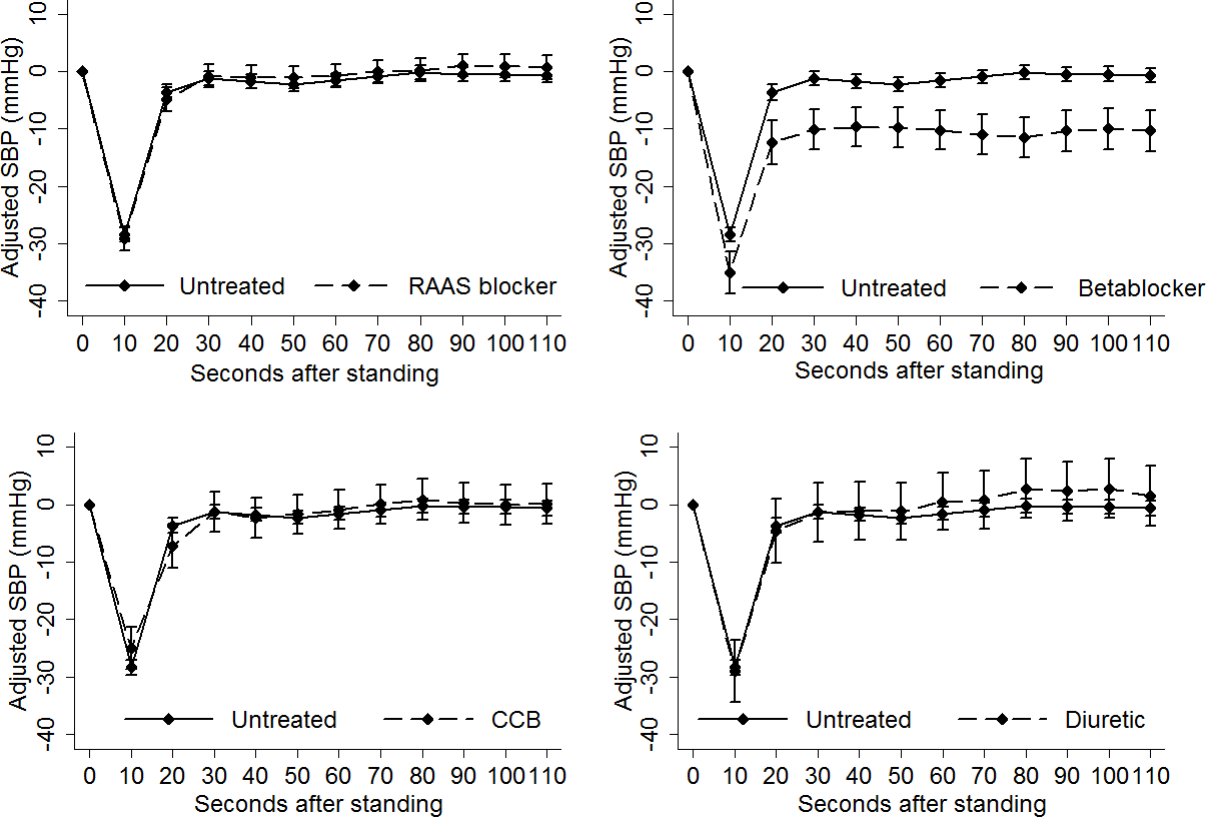
Fully adjusted estimate (95% confidence interval) of mean difference in systolic blood pressure (y-axis) at each 10-second interval during the active stand (x-axis). Each antihypertensive agent was compared to the reference group. The confidence interval for the beta-blocker group did not overlap the point estimate for the reference group. The confidence intervals for the other antihypertensive agents consistently overlapped the point estimate for the reference group. CCB, calcium channel blocker; RAAS, renin-angiotensin-aldosterone-system; SBP, systolic blood pressure.

**Fig 4:**
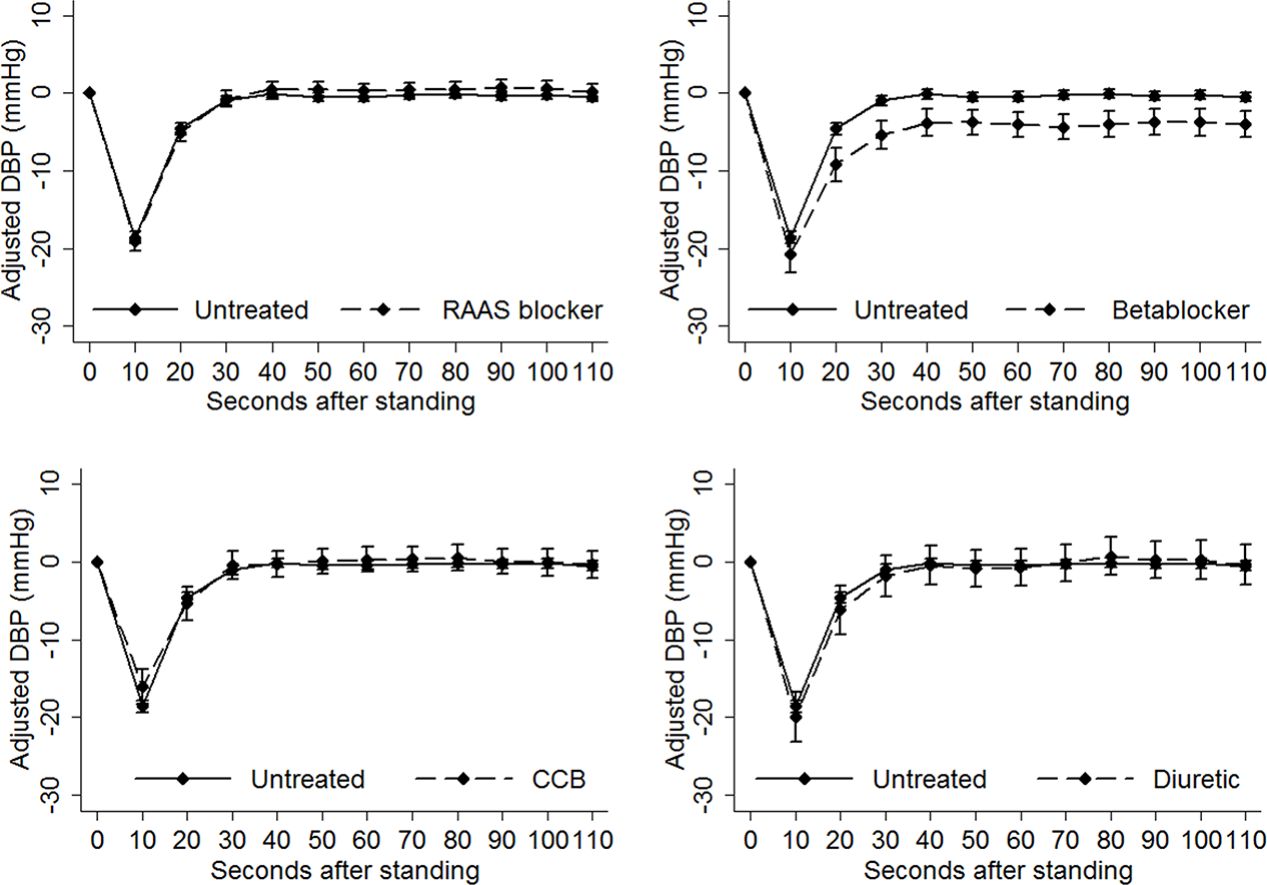
Fully adjusted estimate (95% confidence interval) of mean difference in diastolic blood pressure (y-axis) at each 10-second interval during the active stand (x-axis). As in Fig 2, each antihypertensive agent was compared to untreated grade 1 hypertension. The confidence interval for the beta-blocker group did not overlap the point estimate for the reference group. The confidence intervals for the other antihypertensive agents consistently overlapped the point estimate for the reference group. CCB, calcium channel blocker; DBP, diastolic blood pressure; RAAS, renin-angiotensin-aldosterone-system.

The results of the linear regression analysis are illustrated graphically in **Figs 3 and 4**, showing the fully-adjusted mean difference in systolic **(Fig 3)** and diastolic blood pressure **(Fig 4)** from baseline at each 10-second interval for each antihypertensive drug class and the reference group. For both systolic and diastolic blood pressure, there was no overlap of the error bounds for the beta-blocker group and the point estimate for the reference group for the duration of the active stand, indicating a statistically significant difference in blood pressure between those groups at those time-points. The error bounds for the other three drug classes demonstrated substantial overlap with the reference group.

#### Sensitivity analysis

The logistic regression model for sustained OH was repeated in participants without a history of cardiovascular disease (n = 1175). The results were similar to the previous analysis. Beta-blocker monotherapy was associated with greater than 3-fold increased odds of sustained OH relative to subjects with untreated grade 1 hypertension (unadjusted OR 3.36, 95% CI 1.64–6.91). The result was not attenuated in the fully adjusted model (OR 3.45, 95% CI 1.57–7.59). No signal was detected for the RAAS blocker or calcium channel blocker groups (S2 Table).

#### Secondary analysis

After observing the results of the primary analysis we performed pairwise comparisons of each anti-hypertensive drug class for the outcomes IOH, sustained OH and OH at 30, 60, 90 and 110 seconds. The results are shown in S3 Table. After adjustment for multiple testing, the strongest evidence for a differential effect was observed between beta-blockers and RAAS blockers for all outcomes except IOH. A statistically significant difference was observed between beta-blockers and calcium channel blockers for OH at 30 and 90 seconds. There was insufficient evidence for a difference between beta-blockers and diuretics for any of the above outcomes.

## Discussion

We observed a two-fold increased odds of initial OH and a three-fold increased odds of sustained OH and impaired BP stabilisation in hypertensive older adults receiving beta-blocker monotherapy compared to untreated hypertensive individuals. We did not detect an association between the other major antihypertensive drug classes (RAAS blockers, calcium channel blockers and diuretics) and any of our study outcomes. The effect of beta-blocker therapy was not attenuated after adjustment for potential confounding variables, including the level of baseline supine blood pressure or the presence of cardiovascular disease.

Numerous studies in large populations have demonstrated independent associations between OH and hard adverse outcomes, including all-cause mortality [4, 14], stroke [15], coronary events [16] and heart failure [17]. The link between antihypertensive drug therapy and OH has not been consistent in the literature [18]. Some studies have demonstrated an increased risk of OH with increasing burden of antihypertensive medication [19, 20] but this has not been replicated in all studies [21]. Apart from the potential variability in how OH was measured, prior studies have also varied in terms of gender balance [19, 22] and age profile [23]. In a cross-sectional analysis from the British Women’s Heart and Health Study (BWHHS), Kamaruzzaman et al detected an age-independent association between OH and the use of any BP lowering medication [19]. After adjusting for confounders, the association with beta-blockers remained statistically significant (OR 1.58 [1.19–2.09]). Apart from the fact that this was solely a study of women, a particular challenge in interpreting the results of this study is that not all participants had hypertension and could have been taking beta-blockers for other indications such as underlying coronary artery disease. We found a stronger association between beta-blockers and OH among older adults with a history of self-reported physician-diagnosed hypertension, which persisted even after exclusion of individuals with co-existent cardiovascular disease in a sensitivity analysis.

Romero-Ortuno et al performed a K-means cluster analysis to create three morphological categories of OH using beat-to-beat BP measurements [5]. Membership of the most severe category (“large drop, no recovery”) was associated with the use of beta-blockers and antidepressants. Similar to the BWHHS, the study population was not restricted to those with hypertension, and participants could also have simultaneously been on additional anti-hypertensive medications. It is difficult in the latter context to extrapolate the risk profile for OH associated with an individual anti-hypertensive agent. By selecting a sample of individuals receiving single agent therapy we have further refined the association between antihypertensive agents and OH. In addition we employed the same definitions of OH as those proposed from published normative reference data for beat-to-beat BP measurements [7]. This study adds to the literature by providing a detailed illustration of BP stabilisation during an active stand in a well-defined subpopulation of hypertensive individuals receiving single agent anti-hypertensive therapy.

Recent data suggest that the degree of BP stabilisation after standing, operationalised using beat-to-beat measurements, is an important predictor of clinical outcomes. In a study of older adults referred to an out-patient falls clinic, the trajectory of BP recovery, and not the magnitude of the initial BP decline, predicted adverse outcome. Specifically, failure to recover systolic BP to more than 80% of baseline within 60 seconds of standing was independently associated with a three-fold increased risk of all-cause mortality [8]. Orthostatic hypotension diagnosed using beat-to-beat measurements, rather than auscultatory methods, has been shown to correlate with falls [24]. Previous data from TILDA have demonstrated associations between impaired BP stabilisation and morbidity including cognitive dysfunction [25], frailty [26] and falls [27]. A recurring theme in these studies is the importance of the duration of BP instability and the trajectory of its recovery towards baseline values, rather than the magnitude of the initial fall. The underlying hypothesis is that sustained reductions in BP result in relative hypo-perfusion of vital organs.

Beta-blockers have been a main-stay of anti-hypertensive treatment for several decades, but their efficacy as first-line agents has recently been challenged [28]. A Cochrane review of 13 randomised controlled trials found no difference in all-cause mortality between beta-blockers and placebo, and only a modest reduction in cardiovascular events attributable to beta-blockers [29]. The study also demonstrated that beta-blockers were inferior to other antihypertensive agents with respect to cardiovascular outcomes, and in fact were associated with a higher risk of mortality and cardiovascular disease compared to calcium channel blockers. These findings suggest that beta-blockers should not be a first-line therapy for essential hypertension, not just for lack of efficacy relative to other classes of antihypertensive agent, but also for their potential to do harm.

The mechanism by which beta-blockers could cause impaired BP stabilisation is not clear. Beta-blockers could potentially interfere with several aspects of the compensatory arterial baroreflex arc, such as diminishing the sympathetically-mediated increases in stroke volume, heart rate or peripheral vascular resistance. Such effects could exacerbate orthostatic changes in older adults, who are at higher risk of OH due to a decrease in baroreflex sensitivity that occurs with advancing age [30]. Chronic sustained hypertension is also thought to reduce baroreflex sensitivity, which may in part explain the stronger association we observed with beta-blockers in a nested sample of hypertensive individuals, compared to previous studies which included both hypertensive and non-hypertensive participants [5, 19]. The particular type of beta-blocker may be an important factor in the potential development of OH. For example, nebivolol has been shown to induce a pressor response to orthostasis after 6 months of treatment, an effect that was more pronounced in individuals greater than 60 years of age [31].

Our study has several strengths. Complete beat-to-beat BP data were available in a large number of community-dwelling participants. For each participant their reported list of medications was cross-checked against medication labels and we are confident in the accuracy of ascertainment of medication data among TILDA participants [12]. The dataset is enriched and complete with regard to demographic and health variables. Our study has a number of limitations. As it was an observational study we cannot reliably infer causality in the relationship between beta-blockers and impaired BP stabilisation, nor can we exclude the possibility of residual confounding. The level of ATC coding was such that we did not have data on the particular type of beta-blocker (e.g. cardio-selective or not, half-life) or calcium channel blocker (dihydropyridine or non-dihydropyridine). In addition we did not have information about drug dosage, timing of ingestion or duration of treatment. We did not know the grade or severity of hypertension in the participants prior to them receiving treatment, and it is known that higher baseline blood pressure values are associated with the development of OH [32]. Although we excluded self-reported physician-diagnosed cardiovascular disease in a sensitivity analysis, there remains the possibility that individuals were receiving beta-blockers for secondary prevention of underlying cardiovascular disease, and not solely for the treatment of hypertension. The choice of reference group requires justification. We chose individuals with grade 1 hypertension as our reference category because they represent a group in whom clinicians may be considering starting anti-hypertensive therapy with a single agent. We could not reliably examine effect modification by age due to sample size restrictions. Finally we recorded a single blood pressure response to postural change in each participant and were thus unable to account for potential test-retest variability associated with the use of finometry.

In conclusion, we report an association between beta-blocker monotherapy and orthostatic hypotension during an active stand test in community-dwelling older adults with hypertension, as measured using beat-to-beat technology. Compared to untreated individuals with hypertension, participants receiving beta-blockers demonstrated greater odds of impaired BP stabilisation, a novel outcome measure that is associated with morbidity and mortality in older adults. Future studies should investigate the association between sub-types of beta-blocker and outcomes relevant to an older population such as falls. Our findings lend weight to existing evidence that calls into question the use of beta-blockers as first-line agents for the treatment of essential hypertension.

## Supporting Information

S1 Table Odds ratios (95% confidence intervals) of orthostatic hypotension at each 10-second interval after standing. CCB, calcium channel blocker; RAAS, renin-angiotensin-aldosterone-system. odel adjusted for age, sex, baseline systolic blood pressure, educational attainment, smoking, antidepressant use, other psychotropic medication, diabetes, body mass index, LDL and HDL cholesterol, and cardiovascular disease (any one of angina, myocardial infarction, coronary artery stenting, coronary artery bypass surgery, heart failure, arrhythmia or stroke). †The reference group consisted of untreated participants with grade 1 hypertension.
(DOCX)

S2 Table Logistic regression model showing odds ratios (95% confidence intervals) of sustained orthostatic hypotension after exclusion of participants with pre-existing cardiovascular disease. CB, calcium channel blocker; RAAS, renin-angiotensin-aldosterone-system. Model 1 adjusted for age and sex. Model 2 adjusted for model 1 covariates plus baseline systolic blood pressure. Model 3 adjusted for model 2 co-variates *plus* educational attainment, smoking, antidepressant use, other psychotropic medication, diabetes, body mass index, LDL and HDL cholesterol. † Reference group consisted of untreated participants with grade 1 hypertension. †† No events of sustained OH were encountered in the diuretic group. ** p<0.01
(DOCX)

S3 Table Pairwise comparisons of odds ratios of initial orthostatic hypotension, sustained orthostatic hypotension and orthostatic hypotension at 30, 60, 90 and 110 seconds among anti-hypertensive agents. Each point estimate (and 95% confidence interval) was adjusted for family-wise errors by the Sidak correction. CCB, calcium channel blocker; IOH, initial orthostatic hypotension; OH, orthostatic hypotension; RAAS, renin-angiotensin-aldosterone-system. † No events of sustained OH were observed in the diuretic group. *p<0.05. **p<0.01. ***p<0.001.
(DOCX)

## References

[1] Beckett NS, Peters R, Fletcher AE, Staessen JA, Liu L, Dumitrascu D, et al Treatment of hypertension in patients 80 years of age or older. The New England journal of medicine. 2008;358(18):1887–98. 10.1056/NEJMoa0801369 . 18378519

[2] Benetos A, Labat C, Rossignol P, Fay R, Rolland Y, Valbusa F, et al Treatment With Multiple Blood Pressure Medications, Achieved Blood Pressure, and Mortality in Older Nursing Home Residents: The PARTAGE Study. JAMA internal medicine. 2015;175(6):989–95. 10.1001/jamainternmed.2014.8012 . 25685919

[3] James PA, Oparil S, Carter BL, Cushman WC, Dennison-Himmelfarb C, Handler J, et al 2014 evidence-based guideline for the management of high blood pressure in adults: report from the panel members appointed to the Eighth Joint National Committee (JNC 8). Jama. 2014;311(5):507–20. 10.1001/jama.2013.284427 . 24352797

[4] Ricci F, Fedorowski A, Radico F, Romanello M, Tatasciore A, Di Nicola M, et al Cardiovascular morbidity and mortality related to orthostatic hypotension: a meta-analysis of prospective observational studies. European heart journal. 2015 Epub 2015/04/09. 10.1093/eurheartj/ehv093 . 25852216

[5] Romero-Ortuno R, O'Connell MD, Finucane C, Soraghan C, Fan CW, Kenny RA. Insights into the clinical management of the syndrome of supine hypertension—orthostatic hypotension (SH-OH): the Irish Longitudinal Study on Ageing (TILDA). BMC geriatrics. 2013;13:73 10.1186/1471-2318-13-73 23855394 PMC3716968

[6] Freeman R, Wieling W, Axelrod FB, Benditt DG, Benarroch E, Biaggioni I, et al Consensus statement on the definition of orthostatic hypotension, neurally mediated syncope and the postural tachycardia syndrome. Clinical autonomic research: official journal of the Clinical Autonomic Research Society. 2011;21(2):69–72. 10.1007/s10286-011-0119-5 . 21431947

[7] Finucane C, O'Connell MD, Fan CW, Savva GM, Soraghan CJ, Nolan H, et al Age-related normative changes in phasic orthostatic blood pressure in a large population study: findings from The Irish Longitudinal Study on Ageing (TILDA). Circulation. 2014;130(20):1780–9. 10.1161/CIRCULATIONAHA.114.009831 . 25278101

[8] Lagro J, Schoon Y, Heerts I, Meel-van den Abeelen AS, Schalk B, Wieling W, et al Impaired systolic blood pressure recovery directly after standing predicts mortality in older falls clinic patients. The journals of gerontology Series A, Biological sciences and medical sciences. 2014;69(4):471–8. Epub 2013/07/23. 10.1093/gerona/glt111 . 23873962

[9] Kearney PM, Cronin H, O'Regan C, Kamiya Y, Savva GM, Whelan B, et al Cohort profile: the Irish Longitudinal Study on Ageing. International journal of epidemiology. 2011;40(4):877–84. 10.1093/ije/dyr116 . 21810894

[10] Whelan BJ, Savva GM. Design and methodology of the Irish Longitudinal Study on Ageing. Journal of the American Geriatrics Society. 2013;61 Suppl 2:S265–8. 10.1111/jgs.12199 . 23662718

[11] WHO Collaborating Centre for Drug Statistics Methodology, Guidelines for ATC classification and DDD assignment 2012. Oslo, 2011.

[12] Richardson K, Kenny RA, Peklar J, Bennett K. Agreement between patient interview data on prescription medication use and pharmacy records in those aged older than 50 years varied by therapeutic group and reporting of indicated health conditions. Journal of clinical epidemiology. 2013;66(11):1308–16. 10.1016/j.jclinepi.2013.02.016 . 23968693

[13] Mancia G, Fagard R, Narkiewicz K, Redon J, Zanchetti A, Bohm M, et al 2013 ESH/ESC Guidelines for the management of arterial hypertension: the Task Force for the management of arterial hypertension of the European Society of Hypertension (ESH) and of the European Society of Cardiology (ESC). Journal of hypertension. 2013;31(7):1281–357. 10.1097/01.hjh.0000431740.32696.cc . 23817082

[14] Xin W, Lin Z, Mi S. Orthostatic hypotension and mortality risk: a meta-analysis of cohort studies. Heart (British Cardiac Society). 2014;100(5):406–13. Epub 2013/07/24. 10.1136/heartjnl-2013-304121 . 23878177

[15] Eigenbrodt ML, Rose KM, Couper DJ, Arnett DK, Smith R, Jones D. Orthostatic hypotension as a risk factor for stroke: the atherosclerosis risk in communities (ARIC) study, 1987–1996. Stroke; a journal of cerebral circulation. 2000;31(10):2307–13. . 11022055 10.1161/01.str.31.10.2307

[16] Fedorowski A, Stavenow L, Hedblad B, Berglund G, Nilsson PM, Melander O. Orthostatic hypotension predicts all-cause mortality and coronary events in middle-aged individuals (The Malmo Preventive Project). European heart journal. 2010;31(1):85–91. 10.1093/eurheartj/ehp329 . 19696189 PMC2800919

[17] Fedorowski A, Engstrom G, Hedblad B, Melander O. Orthostatic hypotension predicts incidence of heart failure: the Malmo preventive project. Am J Hypertens. 2010;23(11):1209–15. 10.1038/ajh.2010.150 . 20651699

[18] Zia A, Kamaruzzaman SB, Tan MP. Blood pressure lowering therapy in older people: Does it really cause postural hypotension or falls? Postgraduate medicine. 2015;127(2):186–93. 10.1080/00325481.2015.996505 . 25622817

[19] Kamaruzzaman S, Watt H, Carson C, Ebrahim S. The association between orthostatic hypotension and medication use in the British Women's Heart and Health Study. Age and ageing. 2010;39(1):51–6. 10.1093/ageing/afp192 . 19897539

[20] Heitterachi E, Lord SR, Meyerkort P, McCloskey I, Fitzpatrick R. Blood pressure changes on upright tilting predict falls in older people. Age and ageing. 2002;31(3):181–6. . 12006306 10.1093/ageing/31.3.181

[21] Hiitola P, Enlund H, Kettunen R, Sulkava R, Hartikainen S. Postural changes in blood pressure and the prevalence of orthostatic hypotension among home-dwelling elderly aged 75 years or older. Journal of human hypertension. 2009;23(1):33–9. 10.1038/jhh.2008.81 . 18650837

[22] Poon IO, Braun U. High prevalence of orthostatic hypotension and its correlation with potentially causative medications among elderly veterans. Journal of clinical pharmacy and therapeutics. 2005;30(2):173–8. 10.1111/j.1365-2710.2005.00629.x . 15811171

[23] Valbusa F, Labat C, Salvi P, Vivian ME, Hanon O, Benetos A, et al Orthostatic hypotension in very old individuals living in nursing homes: the PARTAGE study. Journal of hypertension. 2012;30(1):53–60. 10.1097/HJH.0b013e32834d3d73 . 22080223

[24] van der Velde N, van den Meiracker AH, Stricker BH, van der Cammen TJ. Measuring orthostatic hypotension with the Finometer device: is a blood pressure drop of one heartbeat clinically relevant? Blood pressure monitoring. 2007;12(3):167–71. 10.1097/MBP.0b013e3280b083bd . 17496466

[25] Frewen J, Finucane C, Savva GM, Boyle G, Kenny RA. Orthostatic hypotension is associated with lower cognitive performance in adults aged 50 plus with supine hypertension. The journals of gerontology Series A, Biological sciences and medical sciences. 2014;69(7):878–85. 10.1093/gerona/glt171 . 24214492

[26] O’Connell MD, Savva GM, Finucane C, Romero-Ortuno R, Fan CW, Soraghan C, et al Frailty is associated with deficits in early blood pressure recovery post standing in older adults. J Frailty Aging. 2014;3(1):Supplement.

[27] Finucane C, Fan CW CS, O'Connell MDL, Donoghue O, Cronin H, Savva GM, et al Impaired Orthostatic Blood Pressure Control Is Associated With Falls In Community Dwelling Adults Aged Over 50: Findings From The Irish Longitudinal Study On Ageing. Age and ageing. 2014;43 (Suppl 2):ii17 10.1093/ageing/afu130.3

[28] De Caterina AR, Leone AM. Why beta-blockers should not be used as first choice in uncomplicated hypertension. The American journal of cardiology. 2010;105(10):1433–8. 10.1016/j.amjcard.2009.12.068 . 20451690

[29] Wiysonge CS, Bradley HA, Volmink J, Mayosi BM, Mbewu A, Opie LH. Beta-blockers for hypertension. The Cochrane database of systematic reviews. 2012;11:CD002003 10.1002/14651858.CD002003.pub4 . 23152211

[30] Monahan KD. Effect of aging on baroreflex function in humans. American journal of physiology Regulatory, integrative and comparative physiology. 2007;293(1):R3–R12. 10.1152/ajpregu.00031.2007 . 17442786

[31] Cleophas TJ, Grabowsky I, Niemeyer MG, Makel WM, van der Wall EE. Paradoxical pressor effects of beta-blockers in standing elderly patients with mild hypertension: a beneficial side effect. Circulation. 2002;105(14):1669–71. Epub 2002/04/10. . 11940545 10.1161/01.cir.0000012745.50229.ac

[32] Fedorowski A, Burri P, Melander O. Orthostatic hypotension in genetically related hypertensive and normotensive individuals. Journal of hypertension. 2009;27(5):976–82. Epub 2009/04/30. . 19402222 10.1097/hjh.0b013e3283279860

