# Supplementary material for: Single Agent Antihypertensive Therapy and Orthostatic Blood Pressure Behaviour in Older Adults Using Beat-to-Beat Measurements: The Irish Longitudinal Study on Ageing
Source: PLoS One. 2016 Jan 5;11(1):e0146156. doi: 10.1371/journal.pone.0146156 (PMC4701419; doi:10.1371/journal.pone.0146156)
Supplement: S1 Table — CCB, calcium channel blocker; RAAS, renin-angiotensin-aldosterone-system. odel adjusted for age, sex, baseline systolic blood pressure, educational attainment, smoking, antidepressant use, other psychotropic medication, diabetes, body mass index, LDL and HDL cholesterol, and cardiovascular disease (any one of angina, myocardial infarction, coronary artery stenting, coronary artery bypass surgery, heart failure, arrhythmia or stroke). †The reference group consisted of untreated participants with grade 1 hypertension. (DOCX) [file pone.0146156.s001.docx]

|  | **10 sec** | **20 sec** | **30 sec** | **40 sec** | **50 sec** | **60 sec** | **70 sec** | **80 sec** | **90 sec** | **100 sec** | **110 sec** |
| --- | --- | --- | --- | --- | --- | --- | --- | --- | --- | --- | --- |
| **Untreated†** | *ref* | *ref* | *ref* | *ref* | *ref* | *ref* | *ref* | *ref* | *ref* | *ref* | *ref* |
| **RAAS blocker** | 0.95  (0.63-1.44) | 1.24  (0.89-1.74) | 0.94  (0.63-1.42) | 0.69  (0.44-1.09) | 0.83  (0.53-1.31) | 0.77  (0.49-1.22) | 0.81  (0.51-1.30) | 0.93  (0.58-1.49) | 0.83  (0.51-1.36) | 0.89  (0.55-1.45) | 0.67  (0.41-1.11) |
| **Beta-blocker** | 1.23  (0.61-2.49) | 2.59  (1.58-4.25) | 2.98  (1.74-5.11) | 2.46  (1.41-4.28) | 3.07  (1.76-5.37) | 3.18  (1.83-5.53) | 3.45  (1.98-6.0) | 3.12  (1.78-5.48) | 3.23  (1.82-5.74) | 3.22  (1.82-5.72) | 2.90  (1.64-5.11) |
| **CCB** | 0.68  (0.37-1.21) | 1.06  (0.64-1.74) | 0.82  (0.45-1.51) | 0.97  (0.53-1.79) | 0.81  (0.42-1.56) | 0.91  (0.49-1.72) | 0.74  (0.37-1.49) | 0.84  (0.42-1.70) | 0.76  (0.37-1.57) | 0.92  (0.46-1.84) | 0.78  (0.39-1.57) |
| **Diuretic** | 1.34  (0.50-3.58) | 1.66  (0.82-3.37) | 1.32  (0.57-3.04) | 0.90  (0.35-2.35) | 1.20  (0.49-2.98) | 1.13  (0.46-2.77) | 0.13  (0.02-0.98) | 0.75  (0.24-2.30) | 0.74  (0.24-2.29) | 0.56  (0.16-1.95) | 0.69  (0.23-2.12) |
