# Supplementary material for: Single Agent Antihypertensive Therapy and Orthostatic Blood Pressure Behaviour in Older Adults Using Beat-to-Beat Measurements: The Irish Longitudinal Study on Ageing
Source: PLoS One. 2016 Jan 5;11(1):e0146156. doi: 10.1371/journal.pone.0146156 (PMC4701419; doi:10.1371/journal.pone.0146156)
Supplement: S2 Table — CB, calcium channel blocker; RAAS, renin-angiotensin-aldosterone-system. Model 1 adjusted for age and sex. Model 2 adjusted for model 1 covariates plus baseline systolic blood pressure. Model 3 adjusted for model 2 co-variates plus educational attainment, smoking, antidepressant use, other psychotropic medication, diabetes, body mass index, LDL and HDL cholesterol. † Reference group consisted of untreated participants with grade 1 hypertension. †† No events of sustained OH were encountered in the diuretic group. ** p<0.01 (DOCX) [file pone.0146156.s002.docx]

|  | **Unadjusted** | **Model 1** | **Model 2** | **Model 3** |
| --- | --- | --- | --- | --- |
| **Untreated†** | *ref* | *ref* | *ref* | *ref* |
| **RAAS blocker** | 0.84 (0.47-1.50) | 0.78 (0.43-1.39) | 0.88 (0.48-1.59) | 0.84 (0.42-1.67) |
| **Beta-blocker** | 3.36 (1.64-6.91)** | 3.24 (1.55-6.75)** | 3.15 (1.47-6.76)** | 3.45 (1.57-7.59)** |
| **CCB** | 1.24 (0.55-2.84) | 0.99 (0.43-2.30) | 0.81 (0.33-1.96) | 0.90 (0.36-2.22) |
| **Diuretic††** | n/a | n/a | n/a | n/a |
