# Supplementary material for: Single Agent Antihypertensive Therapy and Orthostatic Blood Pressure Behaviour in Older Adults Using Beat-to-Beat Measurements: The Irish Longitudinal Study on Ageing
Source: PLoS One. 2016 Jan 5;11(1):e0146156. doi: 10.1371/journal.pone.0146156 (PMC4701419; doi:10.1371/journal.pone.0146156)
Supplement: S3 Table — Each point estimate (and 95% confidence interval) was adjusted for family-wise errors by the Sidak correction. CCB, calcium channel blocker; IOH, initial orthostatic hypotension; OH, orthostatic hypotension; RAAS, renin-angiotensin-aldosterone-system. † No events of sustained OH were observed in the diuretic group. *p<0.05. **p<0.01. ***p<0.001. (DOCX) [file pone.0146156.s003.docx]

| **Comparison** | **IOH** | **Sustained OH** | **OH(30)** | **OH(60)** | **OH(90)** | **OH(110)** |
| --- | --- | --- | --- | --- | --- | --- |
| Beta-blocker vs RAAS blocker | 1.63  (0.80 – 3.31) | 3.98**  (1.27 – 12.46) | 2.92**  (1.27 – 6.73) | 3.87***  (1.62 – 9.29) | 3.46**  (1.38 – 8.67) | 4.38***  (1.65 – 11.60) |
| CCB vs RAAS blocker | 0.75  (0.34 – 1.65) | 1.36  (0.36 – 5.14) | 0.84  (0.33 – 2.13) | 1.43  (0.54 – 3.81) | 0.98  (0.32 – 2.98) | 1.29  (0.42 – 3.97) |
| Diuretic vs RAAS blocker | 0.72  (0.23 – 2.23) | n/a† | 1.58  (0.47 – 5.28) | 1.72  (0.47 – 6.29) | 1.19  (0.24 – 5.87) | 1.49  (0.28 – 7.77) |
| CCB vs Beta-blocker | 0.46  (0.18 – 1.18) | 0.34  (0.08 – 1.43) | 0.29*  (0.10 - 0.85) | 0.37  (0.12– 1.09) | 0.28*  (0.08 - 0.95) | 0.29  (0.09 - 1.01) |
| Diuretic vs Beta-blocker | 0.44  (0.13 – 1.53) | n/a† | 0.54  (0.14 – 2.03) | 0.45  (0.11 – 1.78) | 0.34  (0.06 – 1.84) | 0.34  (0.06 – 1.91) |
| Diuretic vs CCB | 0.96  (0.27 – 3.39) | n/a† | 1.88  (0.48 – 7.36) | 1.20  (0.29 – 4.96) | 1.22  (0.21 – 6.99) | 1.15  (0.20 – 6.79) |
